# *Rhodobacterales* and *Rhizobiales* Are Associated With Stony Coral Tissue Loss Disease and Its Suspected Sources of Transmission

**DOI:** 10.3389/fmicb.2020.00681

**Published:** 2020-04-23

**Authors:** Stephanie M. Rosales, Abigail S. Clark, Lindsay K. Huebner, Rob R. Ruzicka, Erinn M. Muller

**Affiliations:** ^1^Cooperative Institute for Marine and Atmospheric Studies, University of Miami, Miami, FL, United States; ^2^Atlantic Oceanographic and Meteorological Laboratory, National Oceanic and Atmospheric Administration, Miami, FL, United States; ^3^Mote Marine Laboratory, Elizabeth Moore International Center for Coral Reef Research & Restoration, Summerland Key, FL, United States; ^4^Fish and Wildlife Research Institute, Florida Fish and Wildlife Conservation Commission, St. Petersburg, FL, United States; ^5^Mote Marine Laboratory, Sarasota, FL, United States

**Keywords:** sediment, seawater, epidemic, coral reef, Florida Reef Tract, SCTLD

## Abstract

In 2014, Stony Coral Tissue Loss Disease (SCTLD) was first detected off the coast of Miami, FL, United States, and continues to persist and spread along the Florida Reef Tractr (FRT) and into the Caribbean. SCTLD can have up to a 61% prevalence in reefs and has affected at least 23 species of scleractinian corals. This has contributed to the regional near-extinction of at least one coral species, *Dendrogyra cylindrus*. Initial studies of SCTLD indicate microbial community shifts and cessation of lesion progression in response to antibiotics on some colonies. However, the etiology and abiotic sources of SCTLD transmission are unknown. To characterize SCTLD microbial signatures, we collected tissue samples from four affected coral species: *Stephanocoenia intersepta, Diploria labyrinthiformis, Dichocoenia stokesii*, and *Meandrina meandrites*. Tissue samples were from apparently healthy (AH) corals, and unaffected tissue (DU) and lesion tissue (DL) on diseased corals. Samples were collected in June 2018 from three zones: (1) vulnerable (ahead of the SCTLD disease boundary in the Lower Florida Keys), (2) endemic (post-outbreak in the Upper Florida Keys), and (3) epidemic (SCTLD was active and prevalent in the Middle Florida Keys). From each zone, sediment and water samples were also collected to identify whether they may serve as potential sources of transmission for SCTLD-associated microbes. We used 16S rRNA gene amplicon high-throughput sequencing methods to characterize the microbiomes of the coral, water, and sediment samples. We identified a relatively higher abundance of the bacteria orders *Rhodobacterales* and *Rhizobiales* in DL tissue compared to AH and DU tissue. Also, our results showed relatively higher abundances of *Rhodobacterales* in water from the endemic and epidemic zones compared to the vulnerable zone. *Rhodobacterales* and *Rhizobiales* identified at higher relative abundances in DL samples were also detected in sediment samples, but not in water samples. Our data indicate that *Rhodobacterales* and *Rhizobiales* may play a role in SCTLD and that sediment may be a source of transmission for *Rhodobacterales* and *Rhizobiales* associated with SCTLD lesions.

## Introduction

In 2014, the outbreak of Stony Coral Tissue Loss Disease (SCTLD) was first documented off Virginia Key, Florida (Miller et al., 2016; Precht et al., 2016; Walton et al., 2018). The spatial spread of SCTLD has been rapid, moving 100 km north and 30 km south from Virginia Key within 1 year (Precht et al., 2016). The disease has continued to spread throughout the Florida Reef Tract (FRT; ongoing as of 2020), making it one of the longest documented coral disease outbreaks in the world. To date, SCTLD affects at least 23 species of reef-building corals and the magnitude of this outbreak has led to a reduction of coral diversity in an already fragile ecosystem (Precht et al., 2016; Walton et al., 2018). The Southeast Florida Coral Reef Evaluation and Monitoring Project (SECREMP) found that across 22 sites in Palm Beach, Broward, and Miami-Dade Counties, there was a loss of 30% coral density and 60% of live coral tissue because of SCTLD (Walton et al., 2018).

Initially implicated as a ‘white plague-like disease or syndrome’ (Miller et al., 2016; Precht et al., 2016; Walton et al., 2018), SCTLD now has its own case definition (Stony Coral Tissue Loss Disease [SCTLD], 2018). Corals affected by SCTLD display focal or multifocal lesions, some of which may be preceded by a band of bleached tissue. Rates of tissue loss are acute to chronic, depending on the coral species or specific colony (Stony Coral Tissue Loss Disease [SCTLD], 2018; Aeby et al., 2019). Corals in the Meandrinidae family, such as *Dichocoenia stokesii* and *Meandrina meandrites*, are especially susceptible to SCTLD and are often the first to become affected by SCTLD (Precht et al., 2016; Stony Coral Tissue Loss Disease [SCTLD], 2018; Aeby et al., 2019). Other species, including many brain corals (e.g., *Diploria labyrinthiformis*), are also highly susceptible, and other species, such as *Stephanocoenia intersepta*, appear to have an intermediate susceptibility (Precht et al., 2016). Consequently, the multiple lesion types of SCTLD, the number of susceptible coral species, the persistence of the outbreak, and the geographical scale affected make this an unprecedented disease.

Like many other coral diseases, the cause and environmental sources of transmission of SCTLD are currently unknown. Preliminary studies indicate that lesion progression can be halted on some coral species with antibiotics (Aeby et al., 2019). Therefore, it is feasible that the pathogen(s) may be bacterial, or that a secondary infection by bacteria may be an important contributor to lesion progression. One study identified five bacteria families associated with SCTLD lesions, but none were found across all four coral species examined (Meyer et al., 2019). Coincident with the initial outbreak of SCTLD, a dredging project at the Port of Miami in 2013–2015 increased sedimentation (Miller et al., 2016; Cunning et al., 2019) and relative turbidity (Rosales et al., 2019) in adjacent reefs. This has led to a controversial hypothesis that sediment is a potential source of transmission of the SCTLD pathogen(s) (Miller et al., 2016; Cunning et al., 2019; Gintert et al., 2019). Initial studies also have indicated that SCTLD is likely contagious and waterborne (Precht et al., 2016; Aeby et al., 2019; Muller et al., 2020). Thus, water may be a vector through which sediment-associated SCTLD pathogen(s) may spread via the Florida current (Precht et al., 2016).

**FIGURE 1:**
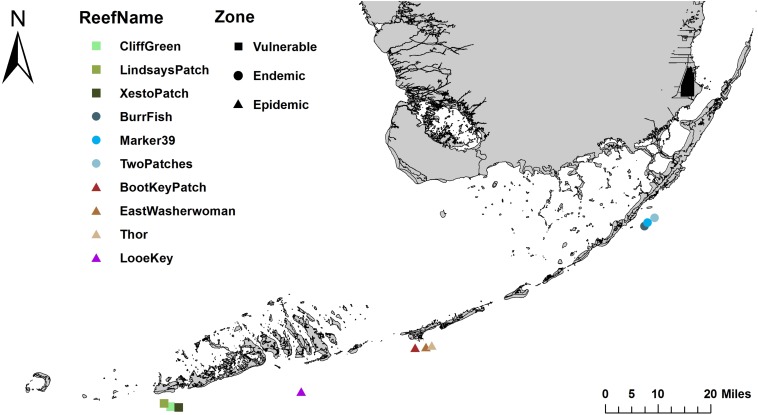
Sample collection sites along the Florida Reef Tract (FRT). Samples were collected at nine mid-channel patch reef sites along the FRT: three sites each in the vulnerable (squares), endemic (circles), and epidemic (triangles) zones. Additional samples were collected at the offshore fore reef Looe Key in the epidemic zone (triangle).

**FIGURE 2:**
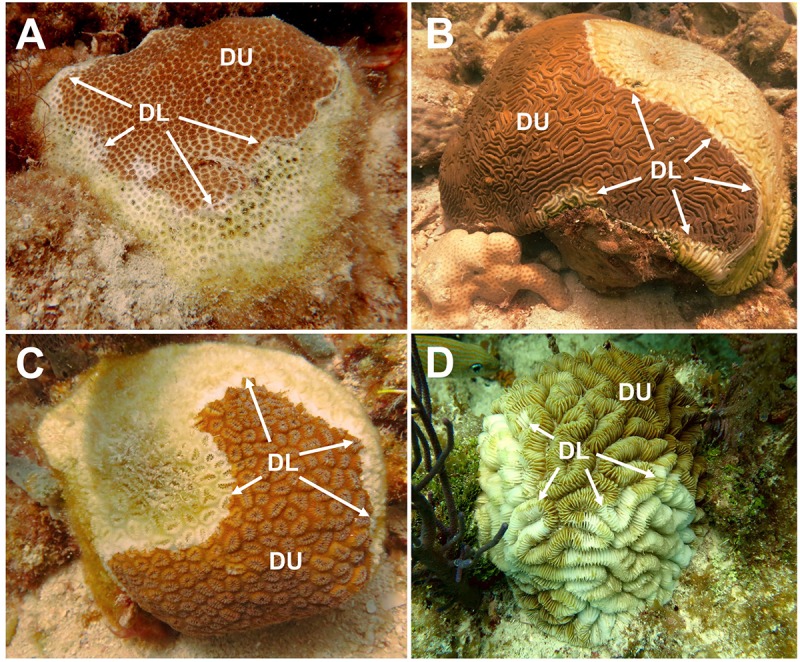
Four coral species sampled for Stony Coral Tissue Loss Disease (SCTLD) microbial analysis. SCTLD tissue samples collected from four species of coral: **(A)** *Stephanocoenia intersepta*, **(B)** *Diploria labyrinthiformis*, **(C)** *Dichocoenia stokesii*, and **(D)** *Meandrina meandrites*. Two tissue samples, unaffected tissue (DU) and lesion margin tissue (DL; denoted by arrows), were collected from colonies showing signs of SCTLD.

The present study investigated the differences among the microbial communities of corals, sediment, and water in the bottom boundary layer. The samples were collected from mid-channel patch reefs within three zones of SCTLD’s geographic progression in the Florida Keys: the vulnerable (pre-invasion; Lower Florida Keys) zone, the endemic (post-outbreak; Upper Florida Keys) zone, and the epidemic (active outbreak; Middle Florida Keys) zone (Table 1 and Figure 1). We sampled coral species with varying levels of susceptibility to SCTLD: *S. intersepta* (intermediate susceptibility), *D. labyrinthiformis* (high susceptibility), and *D. stokesii* (high susceptibility; Figure 2). Coral tissue samples were taken in all three zones and from all three species from apparently healthy (AH) colonies. In the epidemic zone, unaffected tissue on diseased colonies (DU) and lesion tissue (DL) were collected from all three species. A total of five tissue types were collected: (1) vulnerable zone apparently healthy colony (VuAH), (2) endemic zone apparently healthy colony (EnAH), (3) epidemic zone apparently healthy colony (EpAH), (4) epidemic zone diseased colony unaffected tissue (EpDU), and (5) epidemic zone diseased colony lesion tissue (EpDL; Table 1). Additionally, tissue samples from *M. meandrites*, another species with high susceptibility to SCTLD (Precht et al., 2016; Stony Coral Tissue Loss Disease [SCTLD], 2018; Aeby et al., 2019), were collected opportunistically during the onset of SCTLD at Looe Key, an offshore fore reef in the Lower Keys (Table 1 and Figures 1, 2). These samples were included to determine whether the SCTLD-associated microbiomes from this species were consistent with those examined in our original design. In this study, we aimed to: (1) determine if sediment and water in the three SCTLD zones (vulnerable, endemic, and epidemic) showed differences in their microbiomes, (2) determine if AH corals in the three SCTLD zones showed differences in microbiomes, (3) identify microbes associated with SCTLD lesions in four coral species (*S. intersepta, D. labyrinthiformis, D. stokesii*, and *M. meandrites*), (4) identify interactions among microbes associated with SCTLD coral lesions, and (5) determine if microbes associated with SCTLD coral lesions are detected in sediment and water.

**TABLE 1:** Samples analyzed in this study.

Date sampled	Disease outbreak zone	Site name	Number of samples collected				
			Water	Sediment	Apparently healthy colonies (AH)	Diseased colonies x	
						Unaffected tissue (DU)	Lesion margin (DL)
6/28/18	Vulnerable (Vu)	Xesto Patch	10	10			
		Lindsay’s Patch	10	10			
		Cliff Green	10	10	15		
			
			– *Stephanocoenia intersepta* (SINT; *N* = 5), *Diploria labyrinthiformis* (DLAB; *N* = 5), *Dichocoenia stokesii* (DSTO; *N* = 5)				

6/27/18	Endemic (En)	Burr Fish	10	10			
		Marker 39	10	10			
		Two Patches	10	10	15		
			
			– SINT (*N* = 5), DLAB (*N* = 5), DSTO (*N* = 5)				

6/29/18	Epidemic (Ep)	Thor	10	10			
		Boot Key Patch	10	10			
		East Washerwoman	10	10	15	15	15
			
			– SINT (*N* = 10), DLAB (*N* = 10), DSTO (*N* = 10)				

4/19/18	Epidemic (Ep)	Looe Key	3		3	5	5
			
			– *Meandrina meandrites* (MMEA; *N* = 8)				

*Sampling dates, disease zones, site names, and the total number of water (1 L), sediment (5 ml), and coral tissue/mucus slurry (10 ml) samples collected. The coral species, number of colonies sampled per species at each site, and the abbreviations used throughout the manuscript are shown.*

## Materials and Methods

### Sample Collection

Across 3 days in June 2018, samples were collected within the Florida Keys National Marine Sanctuary (FKNMS) from three Stony Coral Tissue Loss Disease (SCTLD) zones, which were assigned based on the geographic progression of SCTLD in the Florida Keys (vulnerable, endemic, and epidemic; Figure 1). All sample sites were mid-channel patch reefs and were chosen based on prior knowledge of the coral community and disease status from the Coral Reef Evaluation and Monitoring Project (CREMP) and/or from scouting immediately prior to sampling. Sites within the endemic zone were scouted before sampling to confirm that no colonies displayed active SCTLD lesions when samples were taken. Sites within the epidemic zone had SCTLD lesions on coral colonies for at least 1 month before our sampling date.

At three sites, within each of the three zones (each site within 5 km of another sampled site; Figure 1), we collected 10 water and 10 sediment samples (30 samples of water and sediment each per zone; Table 1). Water and sediment samples were collected from three sites in each zone to increase sampling replication, but also to account for potential site-specific signatures. Water samples were collected with sterile 1-L Pyrex bottles. The closed bottles were inverted above the substratum, the caps were opened, and the bottles were slowly upturned to allow water to flow in from ∼20 cm above the substratum. Once filled with seawater the bottles were capped. Surface layer sediment samples were scooped directly into 5-ml tubes. In the vulnerable and endemic zones, water and sediment samples were collected near random apparently healthy (AH) corals throughout the reef site, to capture the site-level microbiomes. At epidemic zone sites, water samples were collected above random coral colonies with SCTLD lesions of any species, and sediment samples were collected near these colonies to again capture microbiome signatures at the site level.

At one site in each of the three zones, we collected coral tissue samples from *Stephanocoenia intersepta* (SINT), *Diploria labyrinthiformis* (DLAB), and *Dichocoenia stokesii* (DSTO; Table 1 and Figure 2). These species were selected as the target coral species based on colony availability among the three zones and range of susceptibility to SCTLD. Coral colonies were photographed, measured, and assessed for condition before sampling. Coral samples were collected by scraping 10-ml plastic blunt tip syringes on a small area of the coral surface, and simultaneously pulling the syringe plunger until the syringe was full of a slurry consisting of coral tissue and mucus. Within the vulnerable and endemic zone sites, one sample was taken from five AH coral colonies of each of the three target species (15 samples per zone; Table 1). Within the epidemic zone site, one sample was taken from five AH colonies, and two samples were taken from five diseased colonies, of each of the three target species (45 samples in the epidemic zone; Table 1). The two samples from diseased colonies consisted of one sample from the disease lesion margin (DL) and one sample from unaffected tissue at a distance away from the lesion margin (DU; Figure 2). Thus, we collected five tissue types (Table 1): vulnerable zone apparently healthy colony (VuAH), endemic zone apparently healthy colony (EnAH), epidemic zone apparently healthy colony (EpAH), epidemic zone diseased colony unaffected tissue (EpDU), and epidemic zone diseased colony lesion margin tissue (EpDL). New gloves were worn by the sampler for each colony sampled, and unaffected tissue on diseased colonies was sampled before lesion margin tissue.

After collections, on the boat, coral slurry samples were transferred from the syringes to 15-ml plastic tubes. Tubes with coral tissue, water sample bottles, and sediment sample vials were placed in a dark cooler on ice for transport back to the South Florida Regional Laboratory in Marathon, FL, United States. There, tubes with coral slurry and sediment vials were flash-frozen in a liquid nitrogen dewar. Water samples were filtered through 0.2-μm filters. Next, each filter was placed in a 2-oz Whirl-Pak and flash-frozen in a liquid nitrogen dewar. All samples were transferred to a −80°C freezer at Mote Marine Laboratory in Summerland Key, FL for storage until DNA extractions.

For comparison to the aforementioned epidemic zone samples, *Meandrina meandrites* (MMEA; EpAH, EpDU, and EpDL tissue types) and water (*N* = 3) samples were processed from opportunistic collections in April 2018 at the offshore fore reef Looe Key (Table 1 and Figures 1, 2). These samples were collected within days/weeks of the arrival of SCTLD at this site and MMEA constituted the majority of colonies displaying lesions at the time of sampling. The sampling protocols for coral tissue and water samples were consistent for all sample collections. A summary of the entire sampling effort is detailed in Table 1.

### Sample Processing

All DNA extractions were performed with DNeasy PowerSoil Kits (QIAGEN, Germantown, MD, United States) with modifications to the manufacturer’s protocol. To prepare coral tissue and mucus slurry samples for DNA extractions, the samples were removed from -80°C and thawed at room temperature (20–25°C). Samples were then placed immediately at 4°C. Next, 200 μl of solution were discarded from each DNeasy PowerBead tube, and the beads and remaining solution were transferred to 2 ml microcentrifuge tubes for temporary storage. Coral samples were vortexed for 5 s and 2 ml of every sample were added to the now empty PowerBead tubes. The PowerBead tubes and their contents were then centrifuged for 10 min at 11,000 *g*. The supernatants were carefully removed from the PowerBead tubes and discarded before re-vortexing the coral samples and transferring an additional 2 ml to their respective PowerBead tubes. The tubes and their contents were centrifuged again. This process was repeated until a total of 6 ml of every coral sample had been centrifuged. After removing the supernatants, the PowerBead solutions (in the 2 ml microcentrifuge tubes) were returned to their original tubes on top of the pellets and vortexed for 5 s.

Prior to isolating DNA from the water and sediment samples, 200 μl of the PowerBead solutions were removed and discarded from each respective PowerBead tube. Next, the water filters and sediment samples were removed from −80°C and thawed at room temperature (20–25°C). Half of every 0.2 μm water filter was cut into pieces and added to its respective PowerBead tube. Once thawed, 0.25 g of each sediment sample was added to its respective PowerBead tube. If layers of water were present on top of the sediment then the water was decanted before transferring the sediment.

For all DNA extractions, 200 μl of phenol:chloroform:isoamyl alcohol (pH 7–8) and 60 μl of Solution C1 were added to each sample (coral, water, and sediment). After inverting the samples several times, they were vortexed for 10 min and centrifuged at 10,000 *g*. The supernatants were transferred to new tubes to which 100 μl of Solution C2 and 100 μl of Solution C3 were added. The samples were then vortexed for 5 s and incubated at 4°C for 5 min. Next, the samples were centrifuged for 1 min at 10,000 *g* and the lysates (∼650 μl) were transferred to new tubes. After shaking Solution C4, equal parts of Solution C4 and 100% ethanol were added to the lysates and vortexed for 5 s. Up to 650 μl of lysate were loaded onto each spin column and centrifuged for 1 min at 10,000 *g*. The flow-throughs were discarded, and these steps were repeated until all lysates were loaded and centrifuged. The filter membranes (of the spin columns) were then washed with 650 μl of 100% ethanol and centrifuged for 1 min at 10,000 *g*. Next, the flow-throughs were discarded and 500 μl of Solution C5 were added to the membrane before centrifuging again for 1 min at 10,000 *g*. After discarding the flow-throughs from Solution C5, the membranes were centrifuged and dried for 2 min at 10,000 *g*. The spin columns were then transferred to clean tubes and 60 μl of Solution C6 were added to the membranes. After incubating at room temperature for 5 min, the lysates were centrifuged for 30 s at 10,000 *g* and the flow-throughs (i.e., DNA) were stored at −80°C. DNA yield and quality were evaluated using a NanoDrop One^TM^ Microvolume UV-Vis Spectrophotometer.

The 515F-806R 16S rRNA gene variable region 4 (V4) PCR primers (Apprill et al., 2015) and 1 μl of DNA were used in a single-step 30-cycle PCR using the HotStarTaq Plus Master Mix Kit (QIAGEN, Germantown, MD, United States) under the following conditions: 94°C for 3 min, followed by 30 cycles of 94°C for 30 s, 53°C for 40 s and 72°C for 1 min. After, a final elongation step at 72°C for 5 min was performed. Library preparation and sequencing was performed at MR DNA^1^ (Shallowater, TX, United States) on six lanes of a MiSeq following the manufacturer’s guidelines.

### Bioinformatics Analysis of Amplicon Sequences

Illumina adapters and barcodes were trimmed using MR DNA Free software Application^2^. Trimmed reads were imported into qiime2-2018.11 (Bolyen et al., 2019). The samples were sequenced on six MiSeq sequencing lanes and each lane was initially processed independently. Each sequencing run was demultiplexed using the function qiime demux emp-paired, and forward and reverse primers were removed with cutadapt (Martin, 2011). The data were visualized using the function qiime demux summarize to assess the quality of the reads. Each of the sequence runs showed similar quality, so they were processed the same way with the DADA2 pipeline (Callahan et al., 2016); parameters were –p-trunc-len-f 200, –p-trunc-len-r 200, and maxEE = 2. DADA2 combines identical reads, identifies sequence variants, merges paired-end reads, and removes chimeras, which clusters sequences into amplicon sequence variants (ASVs). The DADA2 output from the six sequencing runs were then merged into one ASV table (count table) and sequence file. ASVs were assigned taxonomy using the function qiime feature-classifier classify-sklearn, a naïve Bayes machine learning classifier (Bokulich et al., 2018). A pre-trained SILVA-132-99-515-806 database was used as the reference database. Upon assigning taxonomy to ASVs, sequences with mitochondria or chloroplast annotations were removed and the reads that were annotated to bacteria or archaea were selected.

### Beta-Diversity Analysis

To evaluate overall beta-diversity, the data were analyzed with the package VEGAN 2.5.4 on R.3.5.1 (Dixon, 2003). The ASV filtered count table was parsed by: sediment, water, each coral species (including all tissue types i.e., VuAH, EnAH, EpAH, EpDU, and EpDL), and each coral species and only AH tissue. Each parsed table was independently transformed using centered log-ratio (CLR) with the package microbiome (Lahti et al., 2017). With CLR-transformed values, dissimilarity indices were generated using the VEGAN function vegdist and tested for homogeneity of group dispersion with betadisper using a Euclidean distance. The significance of dispersion was tested with the VEGAN function Permutation test of multivariate homogeneity of groups dispersions (Permutest). Pairwise dispersion comparisons were conducted with a Tukey multiple comparisons test.

Permutational Multivariate Analysis of Variance (PERMANOVA) was used to identify significant differences among groups using CLR-transformed values for each count table. For sediment and water samples, a PERMANOVA for each was used to test for differences among zones, with the nine reef sites nested within their respective zones (Table 1). Differences among the five tissue types (VuAH, EnAH, EpAH, EpDU, and EpDL) and their interactions with coral species were tested using a factorial PERMANOVA. All PERMANOVA analyses were run using the VEGAN function adonis with 999 permutations and using a Euclidean distance. A pairwise group comparison was assessed for zones and tissue conditions with the function pairwise.adonis and *p*-values were adjusted with a Bonferroni correction (Martinez, 2019).

### Differential Abundance Analysis

To analyze microbial differential abundance within each of the three zones (vulnerable, endemic, and epidemic), an analysis of composition of microbiomes (ANCOM, Mandal et al., 2015) was used for each of the sample types (water, sediment, and coral tissue). All ANCOM analyses were run using the function qiime composition ancom, which conducts a CLR transformation. Prior to running all ANCOM tests, a constant of one was added to the ASV counts table to address the zero counts that cannot undergo a log transformation. To remove noise from sediment and water counts, ASVs that were not found overall in at least 10 samples were removed. For water and sediment samples, ANCOM was run with –m-metadata-column “Zone.” The top 100 microbial taxa with the highest W-statistic values (the higher the W, the more taxa an ASV is significantly different against) from ANCOM were selected for further analysis in sediment and water samples. The W-statistic was used since ANCOM does not output *p*-values.

To identify differences in the microbial communities of AH coral colonies before, during, and after SCTLD progression, we tested for differential microbial abundances among zones for each patch reef species (SINT, DLAB, and DSTO). The AH ASV count tables for each coral species were independently filtered to remove ASVs that were not present in at least 4 samples. ANCOM was then run as stated above.

To identify microbes associated with SCTLD, microbial differential abundances among the five coral tissue conditions (VuAH, EnAH, EpAH, EpDU, and EpDL) were tested separately in each of the three coral species (SINT, DLAB, and DSTO). For MMEA, tissue from EpAH, EpDU, and EpDL were tested for microbial differential abundances. ASVs that were not present in at least four samples were removed from the count tables from all four coral species, and ANCOM was run with –m-metadata-column “Condition.” For MMEA, ASVs that had a *W* = 0 were removed from the analysis since these taxa were not significantly different from other ASVs; the top 30 most abundant bacteria were plotted for MMEA.

### Co-occurrence Analysis

From the above ANCOM analysis, 16 of the significantly differentiated ASVs among tissue conditions were selected for a correlation analysis to identify any potential co-occurrence patterns, which may represent microbial interactions contributing to SCTLD. A z-transformation with the microbiome package (Lahti et al., 2017) was applied to ASV counts and then only EpDL samples were selected for correlation analysis. A spearman correlation matrix was generated using the function cor (R stats) and function round (R base). The *p*-values for the correlation matrix were generated with the function corr_pmat from the package ggcorplot 0.1.3 (Kassambara, 2019) and were set to a significance threshold of *p* < 0.05. The correlation matrix was then plotted using ggcorplot on R.

### Random Forest Analysis

To further identify microbes associated with SCTLD, a machine learning approach was used by applying the function qiime sample-classifier classify-samples. To make a more balanced dataset between the two conditions (AH vs. DL), only the VuAH samples were used since these had not encountered SCTLD. Thus, a comparison of the tissue conditions EpDL (*N* = 20 across four species) and VuAH (*N* = 15 across three species) was analyzed. The subset of 35 samples was filtered to remove ASVs not present in at least 10 samples. We used random forest to classify the data set by either EpDL or VuAH using 500 trees, parameter tuning, a test size of 30% of the dataset (30% of the data was used to test the accuracy of the model and 70% of the data was used to build the model), cross-validation, and optimized feature selection (i.e., recursive feature elimination is used to select a subset of important ASVs to build the model). The ASVs with the top 100 “important” values were further analyzed.

### Network Analysis

To determine ASVs that co-associated in samples from VuAH and EpDL tissues, we conducted two network analyses with the top 100 ASVs selected by random forest using the R package SpiecEasi 1.0.5 (Kurtz et al., 2015). The model selected was the Stability Approach to Regularization Selection (StARS, Han et al., 2010) with the Meinshausen-Bühlmann’s neighborhood selection method (Meinshausen and Bühlmann, 2006). The variability threshold for StARS was set to 10 × e-3 and 100 subsamples. The network centrality (importance of a node) was evaluated with the package tidygraph 1.2.1. (Pederse, 2019) using functions centrality_degree (the number of adjacent edges, i.e., neighbors) and centrality_edge_betweenness (the number of shortest paths going through an edge, i.e., centrality (Brandes, 2001). The top 10 “key players” in each network were selected using the package influenceR 0.1.0. (Simon, 2015), which applies an algorithm called “A family of node importance” to identify important nodes (taxa) in each network (Borgatti, 2006).

## Results

### Sediment and Water Samples Showed Distinct Beta-Diversity Patterns Among Zones

**FIGURE 3:**
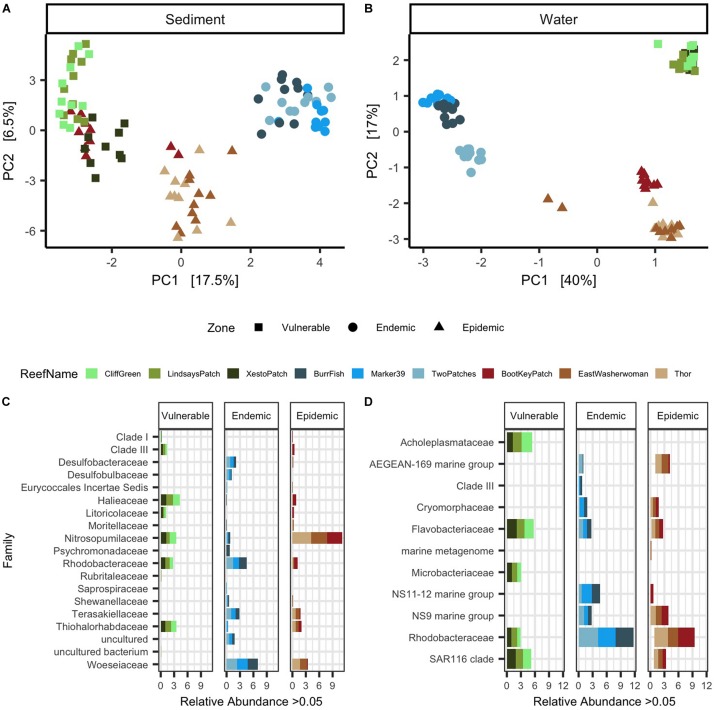
Sediment and water samples showed distinct microbial beta-diversity and taxa among zones. Principal component analysis (PCA) with a Euclidean distance of microbial beta-diversity in **(A)** sediment and **(B)** water samples. Each shape represents a different disease outbreak zone and shapes are colored by reef name. The top differentially abundant taxa among zones are shown in **(C)** sediment and **(D)** water samples. The data are plotted by average percent relative abundances of the most abundant microbial families (>0.05%). Each stacked color bar represents a different reef and each reef is grouped within its respective zone.

In the sediment samples, a total of 2,298 amplicon sequence variants (ASVs) remained after data filtration (frequency per ASV: median = 488, min = 28, and max = 116,193). After filtering water sample data, 468 ASVs remained (frequency per ASV: median = 1,193.5, min = 36, and max = 374,441). Significant differences in microbial beta-diversity dispersion were found among the three SCTLD zones of geographic progression (vulnerable, endemic, epidemic; Table 1 and Figure 1) in both sediment and water samples (*p* < 0.01 from Permutest analyses; Figures 3A,B). A subsequent pairwise comparison of dispersion among zones in sediment samples showed significant differences between the vulnerable-epidemic and endemic-epidemic zone pairings (adjusted *p*-value [padj] = 0.001 and 0.01, respectively), but not between the vulnerable-endemic pairing. In addition, the epidemic zone showed the highest dispersion compared with the other two zones (Figure 3A). For water samples, a pairwise comparison of dispersion of each zone was significant for all comparisons (padj < 0.05; Figure 3B). A PERMANOVA analysis found significant differences in beta-diversity groupings among zones in both sediment (*p* < 0.001, *R*^2^ = 0.31; Figure 3A) and water samples (*p* < 0.001, *R*^2^ = 0.65; Figure 3B).

### Sediment From the Three SCTLD Zones Showed Differently Enriched Microbial ASVs

Microbial differential abundance analysis among the three zones resulted in 136 differently abundant microbial taxa in sediment samples. From the top 100 significantly abundant taxa (according to ANCOM’s W-statistic), different taxa were dominant in each zone (Figure 3C). From the top 100 taxa, the vulnerable reefs had high relative abundances of families *Halieaceae* (mean = 4.4%, *SD* = 1.1%) and *Nitrosopumilaceae* (mean = 3.8%, *SD* = 1.2%). In the endemic reefs, the families *Woeseiaceae* (mean = 7.4%, *SD* = 3.17%) and *Rhodobacteraceae* (mean = 4.8%, *SD* = 1.6%) had the highest relative abundances. The highest relative abundances in the epidemic reefs were from the families *Nitrosopumilaceae* (mean = 11.7%, *SD* = 2.7%) and *Woeseiaceae* (mean = 3.8%, *SD* = 3.0%). Although *Nitrosopumilaceae* was highly prevalent in both epidemic and vulnerable sites, the maximum relative abundance of *Nitrosopumilaceae* in vulnerable sites was 5.9%, but in epidemic sites it was 23.1%. In addition, relative abundances of the entire microbial sediment community also showed that *Nitrosopumilaceae* was relatively more abundant in epidemic sites compared to the other two zones (Supplementary Figure S1A).

### Water From the Three SCTLD Zones Showed Differently Enriched Microbial ASVs

The significance probability from ANCOM found that among the three zones a total of 337 differently abundant microbial taxa were found in water samples. The top 100 differentially abundant taxa present at >0.05% in water samples are shown in Figure 3D. From these taxa, the vulnerable reefs had high relative abundances of the families *Flavobacteriaceae* (mean = 5.8%, *SD* = 4.2%) and *Acholeplasmataceae* (mean = 5.5%, *SD* = 4.8%). The endemic reefs were dominated by the bacterial families *Rhodobacteraceae* (mean = 11.5%, *SD* = 6.1%) and marine group NS11-12 (mean = 4.5%, *SD* = 5.0%). In the epidemic zone, the bacterial families *Rhodobacteraceae* (mean = 9.0%, *SD* = 6.1%) and marine group AEGEAN-169 (mean = 4.1%, *SD* = 6.8%) were the most prevalent. A high prevalence of *Rhodobacteraceae* (mean = 16.7%, *SD* = 3.7%) was also found in opportunistic water samples collected from Looe Key during the beginning of a SCTLD outbreak (Supplementary Figure S1B).

### Microbial Composition of Apparently Healthy Corals From the Same Species Grouped by Zones and Had Differently Enriched ASVs per Zone

**FIGURE 4:**
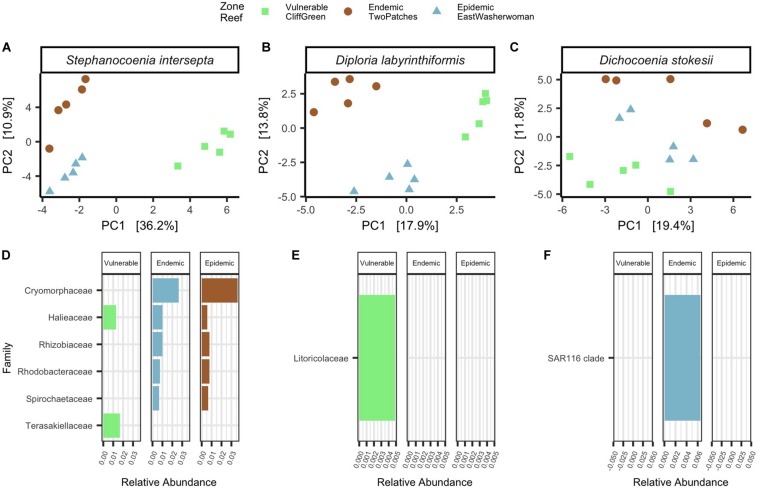
Apparently healthy corals from the same species differed in microbial beta-diversity and taxa by zones. Principal component analysis (PCA) with a Euclidean distance of microbial beta-diversity in apparently healthy **(A)** *Stephanocoenia intersepta*, **(B)** *Diploria labyrinthiformis*, and **(C)** *Dichocoenia stokesii.* Each shape represents a different zone and shapes are colored by reef name. Differentially abundant taxa among zones are shown in apparently healthy **(D)** *S. intersepta*, **(E)** *D. labyrinthiformis*, and **(F)** *D. stokesii.* The data are plotted by average percent relative abundances, colored bars represent different reefs, and each reef is grouped within its respective zone.

To characterize differences in microbial beta-diversity in apparently healthy (AH) corals from the three zones, we collected samples from five colonies of three species per zone (*N* = 45; Table 1). Among filtered count tables, AH *Stephanocoenia intersepta* (SINT) had a total of 1,308 ASVs (frequency per ASV: median = 182, min = 11, and max = 97,961; *N* = 15), AH *Diploria labyrinthiformis* (DLAB) had a total of 439 ASVs (frequency per ASV: median = 205, min = 9, and max = 46,135; *N* = 15), and AH *Dichocoenia stokesii* (DSTO) had a total of 596 ASVs (frequency per ASV: median = 210.5, min = 11, and max = 40,514; *N* = 15). Within the AH tissue samples of each species, a permutest revealed no significant differences in microbial beta-diversity dispersions among zones (Figures 4A–C). However, PERMANOVAs to test significant groupings among zones showed that zone groupings were significant across species (SINT: *p* = 0.001, *R*^2^ = 0.4, Figure 4A; DLAB: *p* = 0.001, *R*^2^ = 0.3, Figure 4B; and DSTO: *p* = 0.002, *R*^2^ = 0.2, Figure 4C). All pairwise comparisons of the three zones in SINT and DLAB were significant (padj < 0.05; Figures 4A,B). For AH DSTO, comparisons of vulnerable-endemic groupings were significant (padj = 0.04), vulnerable-epidemic were nearly significant (padj = 0.07), and endemic-epidemic were not significantly different (Figure 4C).

The ANCOM significance probability analysis found that a total of seven ASVs from six families (*Cryomorphaceae* [ASV 33796], *Halieace* [ASVs 10706 and 20087], *Rhizobiaceae* [ASV 18209], *Rhodobacteraceae* [ASV 29283], *Spirochaetaceae* [ASV 9821], and *Terasakiellaceae* [ASV 29812]) were differentially abundant in AH SINT (Figure 4D). *Cryomorphaceae* had the highest relative abundances in both endemic (mean = 0.14%, *SD* = 0.09%) and epidemic (mean = 0.15%, *SD* = 0.30%; Figure 4F) zones, and an ASV from the bacteria family *Terasakiellaceae* was only present in the vulnerable zone (mean = 0.11%, *SD* = 0.16%; Figure 4D). Differential abundance analysis among the zones of AH DLAB and DSTO resulted in only one bacterial taxon significantly enriched in each coral (Figures 4E,F). For AH DLAB, an ASV from the family *Litoricolaceae* was only present in the vulnerable zone (mean = 0.03%, *SD* = 0.02%; Figure 4E), and AH DSTO had a significant abundance of SAR116 in the endemic zone (mean = 0.15%, *SD* = 0.02%; Figure 4F).

### Coral Samples Differed in Microbial Beta-Diversity by Tissue Condition and Species

To identify microbes associated with SCTLD, a total of 75 samples of five tissue conditions were collected from coral colonies: 15 AH colonies from each zone, and 15 samples of both the unaffected area (DU) and the lesion margin (DL) from diseased colonies in the epidemic zone (Table 1 and Figures 1, 2). After filtering ASVs, there was a total of 1,330 ASVs for SINT (frequency per ASV: median = 205, min = 8, and max = 112,863; *N* = 25), 613 for DLAB (frequency per ASV: median = 189, min = 14, and max = 84,492.0; *N* = 25), and 1,809 for DSTO (frequency per ASV: median = 219, min = 14, and max = 71,127.0; *N* = 25). Coral tissue samples showed distinct groupings from both sediment and water samples (*p* < 0.001, *R*^2^ = 0.2; Supplementary Figure S2). The three coral species also showed distinct groupings by tissue types in their respective zones (vulnerable [Vu] AH, endemic [En] AH, epidemic [Ep] AH, EpDU, and EpDL; *p* < 0.001, *R*^2^ = 0.2) and coral species (*p* < 0.001, *R*^2^ = 0.1) in PCAs (Supplementary Figure S3).

**FIGURE 5:**
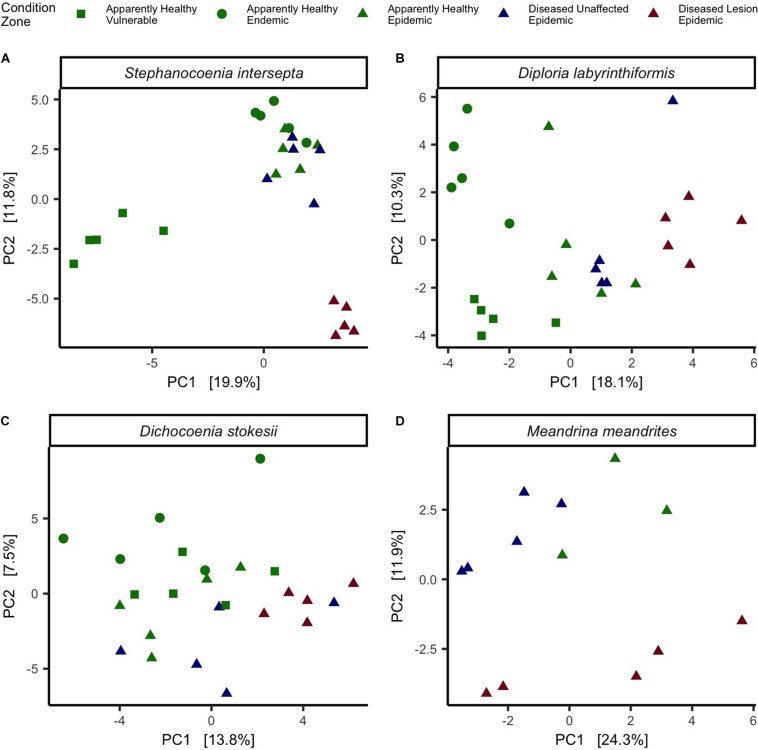
Coral species differed in microbial beta-diversity by tissue condition. Principal component analysis (PCA) with a Euclidean distance of microbial beta-diversity in **(A)** *Stephanocoenia intersepta*, **(B)** *Diploria labyrinthiformis*, **(C)** *Dichocoenia stokesii*, and **(D)** *Meandrina meandrites.* Each shape represents a different zone (vulnerable, endemic, epidemic) and shapes are colored by tissue condition (apparently healthy colony tissue, and unaffected tissue and lesion tissue from a diseased colony).

The three coral species were evaluated separately to identify beta-diversity patterns and differentially abundant microbes that may be associated with SCTLD. In addition, tissue types EpAH, EpDU, and EpDL from *Meandrina meandrites* (MMEA), which were collected opportunistically in the epidemic zone (*N* = 13 samples; Table 1), were included in the analysis. Of the four coral species, only SINT had significant dispersion among the five different tissue types (Permutest = *p* < 0.01, *R*^2^ = 0.4; Figure 5) and a pairwise comparison of dispersion was significant for VuAH-EpDL (padj = 0.0006), EnAH-EpDL (padj = 0.0001), EpAH-EpDL (padj = 0.01), and for EpDU-EpDL (padj = 0.00008; Figure 5A). A PERMANOVA to compare the five tissue types in each species resulted in significant groupings for all four species (*p* < 0.01; Figure 5). A PERMANOVA pairwise comparison of the five tissue conditions was significant in two coral species: in SINT between EpAH-EpDL (padj = 0.05, *R*^2^ = 0.3; Figure 5A) and in DLAB between EnAH-VuAH (padj = 0.05, *R*^2^ = 0.3), EpAH-EpDL (padj = 0.05, *R*^2^ = 0.2), and VuAH-EpDU (padj = 0.04, *R*^2^ = 0.2; Figure 5B).

### The Orders *Rhizobiales* and *Rhodobacterales* Had Significantly Higher Abundances in Lesion Tissue Compared With Other Tissue Types

**FIGURE 6:**
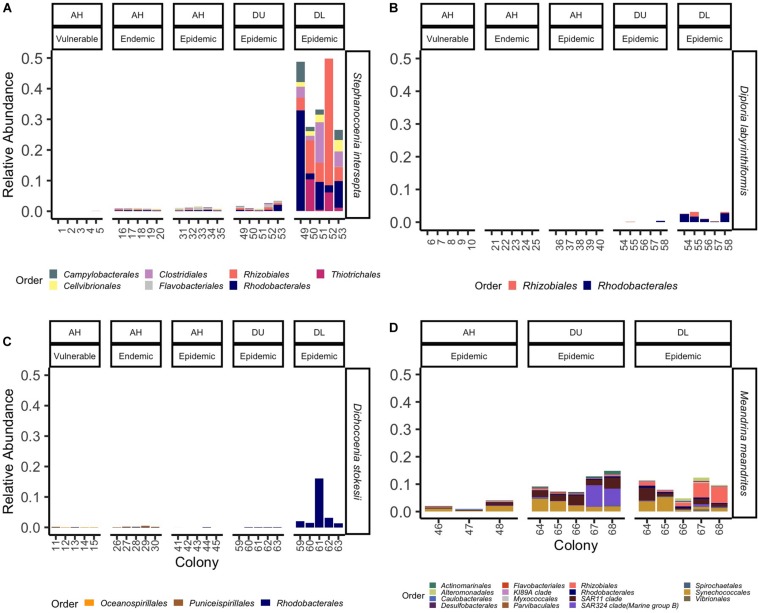
Amplicon sequence variants (ASVs) significantly differentiated in SCTLD samples grouped by bacteria orders. The average relative abundance of significantly differentiated ASVs grouped by order (colors) per coral species **(A)** *Stephanocoenia intersepta*, **(B)** *Diploria labyrinthiformis*, **(C)** *Dichocoenia stokesii*, and **(D)** *Meandrina meandrites.* Samples are grouped by zones (vulnerable, endemic, epidemic) and replicate colonies sampled for three tissue types: apparently healthy colony tissue (AH), and unaffected tissue (DU) and lesion tissue (DL) from a diseased colony.

ANCOM identified a significant probability of differences among the different tissue types in all coral species. For the coral SINT, 12 ASVs were significantly different among the five tissue types. In EpDL SINT samples, the orders *Rhizobiales* (family *Rhizobiaceae*, genus *Cohaesibacter*, ASVs 11394 and 19474; mean range = 0.08–2.4%, *SD* range = 0.17–15.9%) and *Rhodobacterales* (family *Rhodobacteraceae*, ASVs 29283 and 15252; mean range = 0.7–1.4%, *SD* range = 6.5–6.7%) had the highest relative abundances (Figure 6A). Three ASVs were significantly different among the five tissue types in DLAB, one from the order *Rhizobiales* (family *Rhizobiaceae*, genus *Cohaesibacter*, ASV 11394) and two from *Rhodobacterales* (family *Rhodobacteraceae*, ASVs 13497 and 3538). EpDL DLAB samples had the highest relative abundances of both orders *Rhizobiales* (mean = 0.1%, *SD* = 0.6%) and *Rhodobacterales* (mean range = 0.6–0.9%, *SD* = 0.05–0.07; Figure 6B). DSTO had four ASVs that were significantly different among the five tissue types, with two from the order *Rhodobacterales* (family *Rhodobacteraceae*, ASVs 25482 and 2989) found at higher relative abundances in EpDL samples (mean range = 0.2–0.8%, *SD* range = 0.4–6.4%; Figure 6C). After filtering, 75 ASVs were differentiated in MMEA. The top 30 most abundant taxa were plotted, with the exception of the ASVs from the order *Rhodospirillales* (genus *Terasakiellaceae* and AEGEAN-169 marine group) because this order appeared as a dominant member in MMEA across all conditions (AH: mean = 17.4%, *SD* = 20.5%; DU: mean = 18.2%, *SD* = 10.6%; DL: mean = 13.0%, *SD* = 8.5%; Supplementary Figure S1C) and is therefore unlikely to be associated with disease. From the top 29 remaining taxa, EpDL samples had the highest relative abundances of the orders *Synechococcales* (family *Cyanobiaceae*, ASV 15788; mean = 0.8%, SD = 1.9) and *Rhizobiales* (family *Rhizobiaceae*, *Hyphomicrobiaceae*, ASV 24311, and *Stappiaceae*, ASV 19959; mean range = 0.04–0.5%, *SD* range = 0.05-1.2; Figure 6D).

**FIGURE 7:**
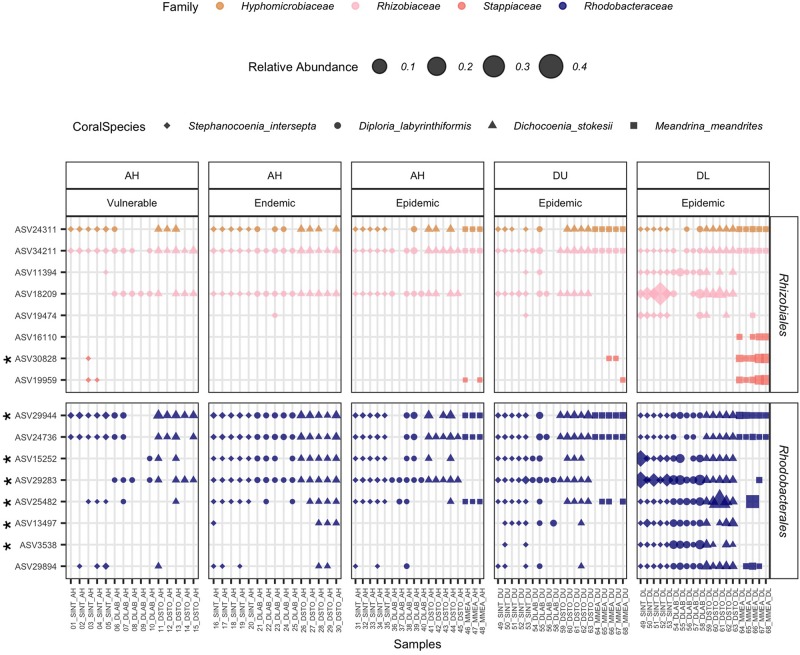
*Rhizobiales* and *Rhodobacterales* enriched in diseased lesions. The relative abundance of significantly differentiated *Rhizobiales* and *Rhodobacterales* amplicon sequence variants (ASVs) per coral sample. On the *x*-axis, samples are grouped by replicate colonies of four coral species (*Stephanocoenia intersepta* [SINT], *Diploria labyrinthiformis* [DLAB], *Dichocoenia stokesii* [DSTO], and *Meandrina meandrites* [MMEA]); three sampled tissue types: apparently healthy colony (AH), and unaffected tissue (DU) and lesion tissue (DL) from diseased colonies; and zone (vulnerable, endemic, epidemic). On the *y*-axis, samples are grouped by bacterial order. The marker size represents the relative abundance of each ASV per sample (scale provided), the shape represents the coral species, and bacterial families are represented by different colors. An asterisk on the ASV name (*y*-axis) denotes that it was selected as “important” in the random forest analysis.

From the four coral species examined, the results of the ANCOM analyses identified significantly higher abundances of the orders *Rhodobacterales* (in 4/4 coral species) and *Rhizibiales* (in 3/4 coral species) in EpDL samples compared with the other tissue types (Figure 6). Thus the 8 *Rhodobacterales* and 8 *Rhizobiales* ASVs that were significantly differentially abundant were further evaluated (Figure 7). The full taxonomic identification along with their sequence information is listed in Supplementary Table S1. Four ASVs (24311, 34211, 29944, and 24736) had higher mean relative abundances in AH samples (mean range RA = 0.07–0.2%) compared to EpDL and EpDU samples. One ASV from *Rhodobacterales* (29894) was present in 90% of EpDL samples and in <35% of all other tissue types. ASVs 3538 from *Rhodobacterales* and 11394 from *Rhizobiales* were present in 93% and 87% of EpDL samples, respectively, and at <13% in the other tissue types in three coral species (SINT, DLAB, DSTO). Three ASVs from *Rhizobiales* (30828, 19959, and 16110) were prevalent in MMEA but were at low prevalence in the other three coral species (Figure 7).

### *Rhodobacterales* Showed Significant Interactions Within the Microbial Community

**FIGURE 8:**
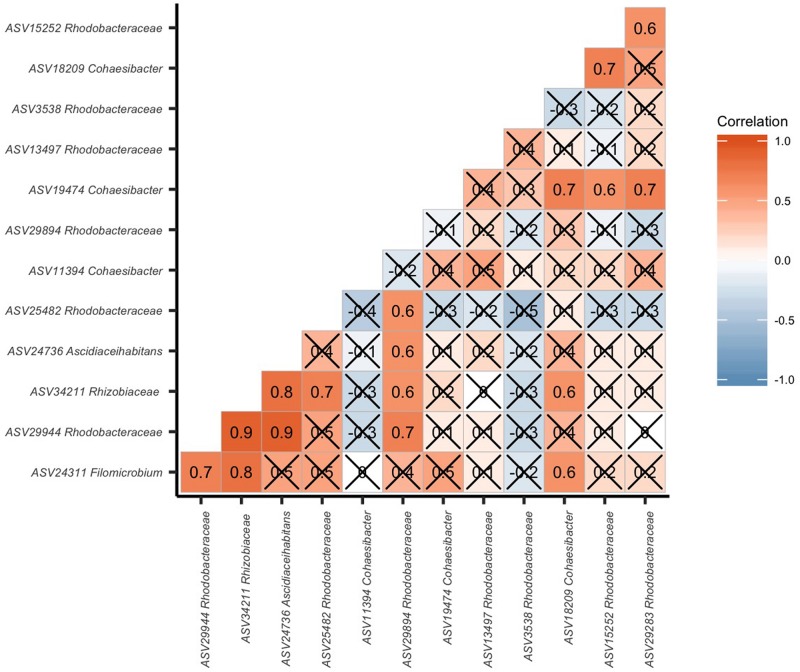
*Rhizobiales* and *Rhodobacterales* are positively correlated in diseased lesions. Pairwise correlation of the 16 significantly differentiated *Rhizobiales* and *Rhodobacterales* amplicon sequence variants (ASVs). The ASVs are labeled to the lowest taxonomic identification. In each block, the number and color denote a correlation value. The higher the correlation, the higher the number and the darker the red coloring. The lower the correlation, the more negative the number and the darker the blue coloring. If boxes are marked with an “X”, the correlation was not significant (*p* > 0.05).

To identify potential interactions in EpDL samples of the 8 *Rhizobiales* and 8 *Rhodobacterales* ASVs, a correlation analysis of these 16 taxa was conducted. There were 17 significant positive correlations among these taxa and no significant negative correlations (Figure 8). The two highest correlations (0.9) occurred between *Rhodobacterales* ASV 29944 versus *Rhizobiales* ASVs 34211 and 24736.

**FIGURE 9:**
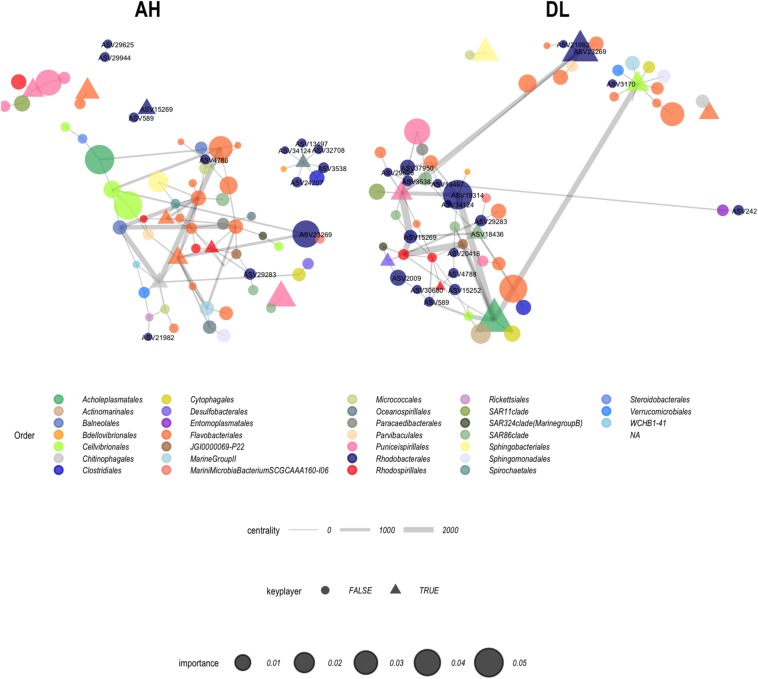
Coral microbiome network analysis. Apparently healthy (AH) samples from the vulnerable zone (*N* = 15) and diseased colony lesion (DL) samples from epidemic zone (*N* = 20) were used to construct networks. Each node represents an amplicon sequence variant (ASV) and is sized based on the random forest importance value (scale provided). Shapes correspond to whether the ASVs were identified as a “key player” and the width of connecting lines denote ASV centrality (scale provided). Only *Rhodobacterales* (navy) nodes are labeled with their ASV numbers.

From the 16 ASVs, seven were “important” in the random forest classification of EpDL and VuAH samples (ASVs marked by asterisks in Figure 7): six ASVs from *Rhodobacterales* (13497, 3538, 29944, 25482, 15252, and 29283) and one ASV from *Rhizobiales* (30828). ASVs with an importance value > 0 are listed in Supplementary Table S2. From these random forest results, the top 100 taxa were evaluated with network analysis to identify interaction structures in VuAH and EpDL samples. Both networks had 68 nodes with 98 edges and were undirected (Figure 9). In VuAH samples, the four taxa with the highest degree of centrality (*N* = 9) were from the order *Flavobacteriales*. In EpDL samples, the highest degree of centrality was from the orders *Puniceispirillales* (*N* = 11), *Cellvibrionales* (*N* = 10), *Rhodospirillales* (*N* = 9), and *Acholeplasmatales* (*N* = 7). From VuAH samples, the orders with “key players” from multiple ASVs were from the orders *Flavobacteriales* (19985, 1345, 29282, and 34847) and *Rhodobacterales* (589 and 2994). EpDL samples had three bacterial orders containing “key players” with multiple ASV representatives: *Flavobacteriales* (14529 and 1899), *Rhodobacterales* (19314 and 23269), and *Rhodospirillales* (36201 and 5342). The order *Rhodobacterales* was less prevalent (13 ASVs) in the VuAH network than in the EpDL network, and only three *Rhodobacterales* had >2 neighbors (and these were not “key players”). In addition, the majority of *Rhodobacterales* (*N* = 9) did not connect to the main network. In contrast, EpDL samples had 18 *Rhodobacterales* ASVs of which 12 had >2 neighbors (neighbor range = 1–6), with all 18 ASVs integrated into the main network. From the seven ASVs found in both random forest and ANCOM analysis, four were part of the EpDL network analysis (29283, 15252, 3538, and 13497, all from *Rhodobacterales*).

### The Orders *Rhizobiales* and *Rhodobacterales* Were Found in Sediment and Water Samples

**FIGURE 10:**
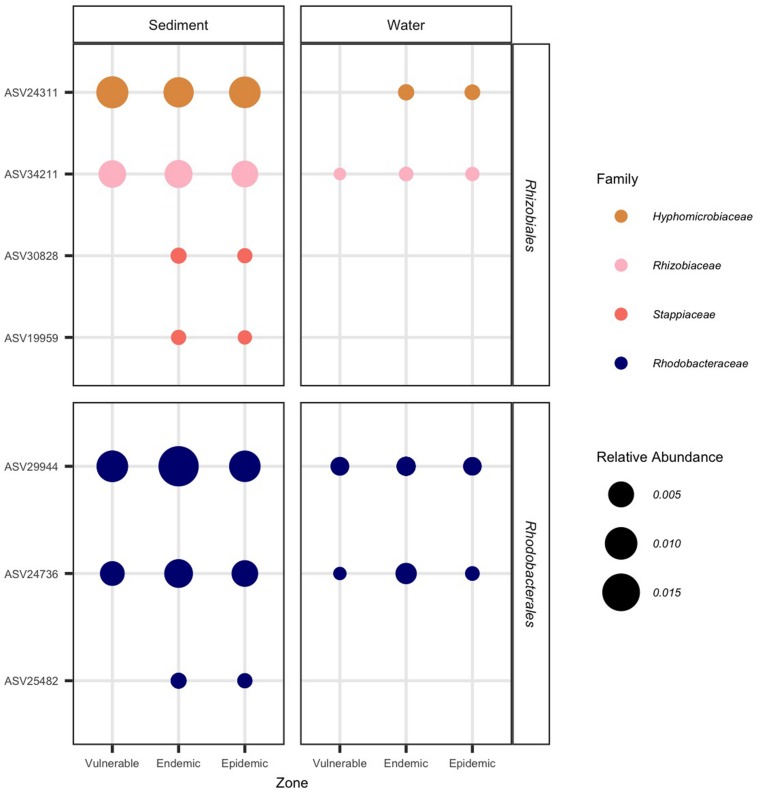
*Rhizobiales* and *Rhodobacterales* found in sediment and water samples that were significantly differentiated in diseased colony lesions. The relative abundance of significantly differentiated *Rhizobiales* and *Rhodobacterales* amplicon sequence variants (ASVs) per zone. On the *x*-axis, samples are grouped by zone and type (sediment or water). On the y-axis, samples are grouped by bacterial order. The marker size represents the relative abundance of each ASV (scale provided) and bacterial families are represented by different colors.

We investigated whether the 16 ASVs from *Rhizobiales* and *Rhodobacterales* were present in the water and sediment samples. Of these 16 ASVs, seven (44%) were found in sediment and four (25%) were found in water samples (Figure 10). Although the four ASVs in the water samples overlapped with those found in the sediment samples, they showed lower relative abundances in the water. In the sediment samples, two ASVs from *Rhizobiales* (30828 and 19959) and one from *Rhodobacterales* (25482) were only found in endemic and epidemic sites, but were absent in vulnerable sites and in all water samples. For water samples, ASV 24311 was detected in the endemic and epidemic zones and not the vulnerable zone, however, it was present across all zones in the sediment samples.

## Discussion

Stony Coral Tissue Loss Disease (SCTLD) is an ongoing multi-year coral disease outbreak first identified in South Florida and now spreading in the Caribbean (Walton et al., 2018; Alvarez-Filip et al., 2019). It affects multiple coral species including US Endangered Species Act listed corals and has led to the regional near-extinction of *Dendrogyra cylindrus* (Stony Coral Tissue Loss Disease [SCTLD], 2018). The severity of the outbreak has led to unprecedented response efforts to mitigate its spread, such as placing wild, apparently healthy (AH) coral colonies in land-based aquaria for safekeeping, and treating diseased colonies with antibiotics *in situ* (Neely et al., 2019). To better understand SCTLD, we conducted 16S rRNA gene high-throughput sequencing of tissues from AH and diseased colonies of four coral species: *Stephanocoenia intersepta* (SINT), *Diploria labyrinthiformis* (DLAB), *Dichocoenia stokesii* (DSTO), and *Meandrina meandrites* (MMEA; Table 1 and Figure 2). Additionally, we sequenced samples from potential environmental transmission sources of SCTLD (sediment and water) from three zones of SCTLD progression (vulnerable [Lower Florida Keys], endemic [Upper Florida Keys], and epidemic [Middle Florida Keys]; Table 1 and Figure 1).

### Potential Microbial Signatures Associated With SCTLD Outbreaks Found in Water and Sediments

Our results showed that sediment and water microbial beta-diversity was significantly different among vulnerable, endemic, and epidemic SCTLD zones (Figure 3). Water and sediments collected from distinct geographical regions can have discrete microbial compositions (Cui et al., 2019; Glasl et al., 2019). Although geographical location may have been the main factor driving site groupings (Glasl et al., 2019), this pattern is intertwined with the unique environmental conditions from each zone (Martiny et al., 2015; Quero et al., 2015). Microbial signatures are routinely used to identify poor environmental conditions for recreational water use (Fong et al., 2005) and, more recently, studies have shown that microbial markers are also indicators of reef health (Glasl et al., 2017, 2019). Therefore, the unique microbiome beta-diversity found in sediment and water samples in epidemic and endemic zones may also be correlated with environmental conditions related to SCTLD outbreaks.

Sediment samples collected from the SCTLD epidemic zone had significantly higher relative abundances of the family *Nitrosopumilaceae* compared to both endemic and vulnerable zones (Figure 3C). *Nitrosopumilaceae* consist of a group of ammonia-oxidizing archaea (Qin et al., 2017) commonly found in marine environments (Gajigan et al., 2018; He et al., 2018) and are positively correlated with high levels of nitrite and nitrate (Gajigan et al., 2018). *Nitrosopumilaceae* is likely not directly responsible for SCTLD, but this group of archaea may signify environmental conditions that promote the prevalence of SCTLD, or could be a secondary response to disease occurrence in the reef. The higher relative abundance of *Nitrosopumilaceae* in the sediment from the epidemic zone may also indicate that there was higher nitrite and nitrate concentrations in the water compared to the other zones. Future studies should consider coupling water quality testing and SCTLD monitoring to examine if there is any relationship between the two.

Microbial communities from reef water may be a better indicator of coral reef health compared with either reef sediments or tissue from AH corals (Glasl et al., 2019). Our analysis of the microbiome in water samples showed that the bacterial family *Rhodobacteraceae* had higher relative abundances in epidemic and endemic zones compared to vulnerable reefs (Figure 3D and Supplementary Figure S1B). High relative abundances of *Rhodobacteraceae* have been found in water samples of reefs in poor health and are suggested to be indicators of stressed reefs (Glasl et al., 2019). Members of *Rhodobacteraceae* also increase in abundance after episodes of poor ocean conditions caused by algal blooms (Lamy et al., 2009). Some members of this family are thought to persist in the environment using suboptimal energy sources and upon availability switch to more favorable energy sources (e.g., the sulfur compound dimethylsulphoniopropionate [DMSP]), thereby increasing in abundance (Durham et al., 2014). DMSP is also found at significant concentrations on coral reefs and *Rhodobacteraceae* found on corals can potentially degrade DMSP (Raina et al., 2010). It is possible that reefs in the epidemic and endemic zones had more favorable energy sources for *Rhodobacteraceae.* However, *Rhodobacteraceae* are widely distributed at high abundances in the ocean and are metabolically diverse (Brinkhoff et al., 2008). Thus, it is unknown if high relative abundances of *Rhodobacteraceae* in water from endemic and epidemic zones are driven by the same factors that led to an SCTLD outbreak, a consequence of SCTLD itself, or just inherent to the reefs sampled. Both *Nitrosopumilaceae* and *Rhodobacteraceae* may be of interest for further research as potential signatures of SCTLD in the environment.

### Apparently Healthy Corals in Vulnerable, Endemic, and Epidemic Zones Showed Differences in Microbial Composition

AH corals from the same species collected from the three zones showed clear groupings by zone in each species (Figures 4A–C). Of the three coral species, overall beta-diversity shifts were more notable in SINT (Figure 4A and Supplementary Figure S3). AH SINT from the vulnerable zone had different bacteria enriched compared with the endemic and epidemic zones (Figure 4D). Unlike in the vulnerable zone, AH SINT in the endemic and epidemic zones had three bacterial taxa that have been linked to coral diseases - including SCTLD: *Cryomorphaceae* (Gignoux-Wolfsohn et al., 2017; Meyer et al., 2019), *Rhizobiaceae* (Cárdenas et al., 2012; Meyer et al., 2019), and *Rhodobacteraceae* (Sunagawa et al., 2009; Cárdenas et al., 2012; Roder et al., 2014; Gignoux-Wolfsohn et al., 2017; Pollock et al., 2017; Meyer et al., 2019) (Figure 4D). Although AH colonies of the other two coral species also grouped by zones, colonies sampled in the epidemic and endemic zones did not show any signatures that would suggest that they were exposed to disease (Figures 4E,F). A coral’s geography and disease susceptibility can correlate to the structure of its microbiome (Pollock et al., 2018; Rubio-Portillo et al., 2018), but we are unaware of any study that has shown how a post-invasion or active invasion of disease on a reef can affect the microbiome of AH corals. The significantly higher levels of *Cryomorphaceae, Rhizobiaceae*, and *Rhodobacteraceae* in AH SINT colonies in the epidemic and endemic zones suggest that these corals may harbor disease-related bacteria without succumbing to tissue loss. The ability of AH SINT to appear healthy with disease associated bacteria in their microbiomes may be why these corals show a lower susceptibility to SCTLD than other species during surveys. This possible resilience to SCTLD of some SINT colonies is an interesting question for future research.

### SCTLD Microbial Signatures in Lesions of Four Diseased Coral Species

Similar to other microbial disease studies, a significant shift in overall beta-diversity occurred between AH and diseased corals in the four coral species examined within the present study (Figure 5). In lesion tissue from diseased colonies in the epidemic zone (EpDL), there was a significantly higher abundance of the orders *Rhodobacterales* (in 4 of 4 coral species) and *Rhizobiales* (in 3 of 4 coral species; Figure 6). Other significant bacterial orders were found but we reason these may be species-specific opportunists or are associated with healthy corals, given that they were only present in one of the four species (Figure 6). Given this pattern, we think that *Rhodobacterales* and *Rhizobiales* may be associated with SCTLD across multiple species and may be of interest for SCTLD research.

Combined, 16 amplicon sequence variants (ASVs) from *Rhodobacterales* and *Rhizobiales* significantly differed among coral tissue types, with a random forest analysis identifying seven of them as “important” (Figure 7 and Supplementary Tables S1, S2). We hypothesize that from the 16 ASVs, the four found at higher relative abundances across AH (ASVs 24311, 34211, 29944, and 24736) compared to EpDL samples were unlikely to be detrimental to the health of the coral. Notably, MMEA showed a distinct pattern of these 16 ASVs. Two *Rhizobiales* ASVs (30828 and 16110) were prevalent in >80% of EpDL MMEA samples, but were absent in both AH MMEA samples and in EpDL samples of the other three coral species. It is possible that the unique microbiome of MMEA, which is dominated by *Rhodospirillales* (Supplementary Figure S1C), may respond differently to SCTLD than other corals. MMEA is one of the species most susceptible to SCTLD, with up to a 98% disease prevalence in reefs, and it is one of the first species to show signs of SCTLD during outbreaks (Precht et al., 2016; Stony Coral Tissue Loss Disease [SCTLD], 2018; Aeby et al., 2019). A disturbance of the dominant *Rhodospirillales* may cause more severe changes in the microbiome of MMEA than in coral species with a low prevalence of this order, or with more than one dominant taxa (Supplementary Figure S1C). For example, we found 75 differentially abundant taxa among epidemic AH, DU, and DL tissues in MMEA; in contrast, the other coral species had a combined total of <12 differentially abundant taxa (Figure 6). However, DSTO is in the same family as MMEA (Meandrinidae) and is also one of the species most impacted by SCTLD, with up to a 97% disease prevalence (Precht et al., 2016). Thus, it is surprising we do not see more similarity between MMEA and DSTO in their microbiome shifts due to SCTLD.

The difference in abundance of *Rhizobiales* and *Rhodobacterales* between coral species may be due to the various differences between when and where the samples were collected (Table 1). The MMEA in the present study were sampled at an offshore fore reef (Looe Key) in April, at the beginning of the SCTLD outbreak at that site. In comparison, the other three coral species were sampled at mid-channel patch reefs in June, during an established SCTLD outbreak (see Methods). Different reef habitats (in particular inner vs. outer reefs) have different rates of disease incidences (Rippe et al., 2019), which may have an effect on coral microbiomes.

A difference in disease stage may also explain some of the variations between these two sampling efforts. *Rhizobiales* may be found at higher abundances at the early stages of SCTLD and *Rhodobacterales* may increase in abundances during later stages. A similar pattern is seen in freshwater microcystic blooms, on the first day *Rhizobiales* are one of the most abundant bacteria orders and after 2–4 days *Rhizobiales* decrease and *Rhodobacterales* become one of the most abundant orders (Li et al., 2011). *Rhodobacterales* are associated with multiple coral diseases, and this may not be because they are causative agents of disease, but rather they may be efficient successors and readily colonize diseased corals (Pollock et al., 2017). For example, corals inoculated with the known coral pathogen *Vibrio coralliilyticus* showed that initial timepoints had high relative abundances of *Vibrionales*, but hours later the relative abundances of *Vibrionales* decreased and relative abundances of *Rhodobacterales* increased (Welsh et al., 2017). A time-series experiment may help determine if there is a succession event between *Rhizobiales* and *Rhodobacterales* during SCTLD infections. Our results also showed that no single *Rhodobacterales* ASV was detected in every EpDL sample (Figure 7), suggesting they are secondary pathogens or opportunistic colonizers. It is also unknown if all SCTLD lesions are derived from the same pathogen(s), which could lead to different bacteria found in lesions. However, to combat this, we attempted to reduce ecological variability in our original study design (SINT, DLAB, DSTO patch reef collections) by sampling from diseased colonies in a single region (Figure 1), with similar appearances of lesion progression (Figure 2), and during the same time frame (Table 1).

### Interactions Between *Rhizobiales* and *Rhodobacterales* in Corals With SCTLD

The presence of *Rhizobiales* and *Rhodobacterales* among coral species suggests that they may associate with one another at some point during disease progression. The co-occurrence analysis showed only positive interactions between *Rhizobiales*-*Rhodobacterales*, *Rhizobiales*-*Rhizobiales*, and *Rhodobacterales*-*Rhodobacterales* (Figure 8). These results may indicate that a consortium of these taxa may be working together during a SCTLD infection. This is similar to black band disease (BBD), which is caused by a consortium of several bacteria taxa (Cooney et al., 2002). While interactions of *Rhizobiales* and *Rhodobacterales* have not been reported in coral diseases, these taxa have co-occurred within colonies showing signs of white plague disease on Caribbean corals *Pseudodiploria strigosa* and *Siderastrea siderea* (Cárdenas et al., 2012) and within BBD on *S. siderea* (Sekar et al., 2006). Alternatively, the presence of both *Rhizobiales* and *Rhodobacterales* in SCTLD lesions may be part of a random co-occurrence event with the coral host (Hester et al., 2016). Thus, these bacteria may co-occur, but not necessarily interact, which are dynamics that are difficult to untangle with single time-point gene marker datasets.

However, *Rhodobacterales* are known to work together in the ocean to form biofilms (Elifantz et al., 2013), and this intrinsic cooperative behavior may aid in disease development or colonization on already diseased tissue (Parsek and Singh, 2003; Pollock et al., 2017). The EpDL network analysis showed multiple *Rhodobacterales* interacting with one another and acting as “key players” of the network (Figure 9). The EpDL network analysis did not show any interactions with *Rhizobiales*, despite their enriched abundance in DL tissues of three species. However, this was not surprising given that the random forest results (the input data used for the network analysis) only selected the *Rhizobiales* associated with MMEA DL samples (Figure 7), and our filtering pipeline removed less prevalent ASVs to reduce noise and to make the classification process more robust.

### *Rhizobiales* and *Rhodobacterales* From SCTLD Lesions Were Found in Sediment and Water Samples

To investigate if *Rhizobiales* and *Rhodobacterales* found in SCTLD lesions can be detected in the water or sediment, we examined water and sediment samples for the 16 significantly differentiated ASVs found in the coral tissue samples (Figure 7). Of the 16, only seven of these ASVs were detected in the sediment samples, of which four were also detected in the water samples (Figure 10). The relative abundances were higher in the sediment samples, suggesting that these bacteria are more concentrated in sediments compared to the water column. This may be because seawater microbes respond more readily to environmental changes compared to sediment microbes (Glasl et al., 2019); in turn, this may result in a higher turnover rate of bacteria in the water column compared to the sediment. The three ASVs that were found only in the sediment samples (from *Rhizobiales*: ASVs 30828 and 19959; from *Rhodobacterales*: ASV 25482) were within the epidemic and endemic sediments and were among the ASVs found at higher abundances in DL and DU coral tissues compared to AH tissue. This indicates that sediment may be a source of transmission for bacteria associated with SCTLD and experiments are needed to understand the full role of sediment in SCTLD outbreaks. The endemic zone was over a year removed from an epidemic status, yet the sediments at these sites still contained SCTLD-associated bacteria. The persistence of these bacteria in site sediments and whether they may be pathogenic to AH corals are additional research priorities. This may be of particular interest to restoration efforts within the endemic zone of species susceptible to SCTLD.

## Conclusion

Overall, this study aimed to identify microbes associated with SCTLD lesions and its suspected sources of transmission. We identified multiple ASVs from *Rhodobacterales* that were correlated with SCTLD in ways such as: (1) a high relative abundance of *Rhodobacterales* in epidemic and endemic water samples, (2) the presence of *Rhodobacterales* in AH SINT colonies from epidemic and endemic sites, (3) the high prevalence of *Rhodobacterales* across EpDL tissue samples from all coral species, and (4) the centrality and high number of interactions of *Rhodobacterales* in the EpDL network analysis. Each result encompassed a unique set of *Rhodobacterales* ASVs, which was not unexpected given the abundance and metabolic diversity of *Rhodobacterales* in the ocean. The adaptability of this bacterial order may be one of the reasons *Rhodobacterales* is often associated with coral diseases.

In our study, *Rhodobacterales* and *Rhizobiales* showed patterns that warrant further investigation into their roles in SCTLD. However, our methods cannot determine if these are the causative agents of SCTLD. While it is still necessary to pinpoint the causative agent of SCTLD, it would also be worth examining pathways that can target and halt the replication of both *Rhodobacterales* and *Rhizobiales* to determine if these actions mitigate SCTLD disease progression. Lastly, *Rhodobacterales* and *Rhizobiales* ASVs found in EpDL coral tissue were also detected in water and sediment, providing some evidence that these environments may be sources of transmission for SCTLD-associated bacteria. The higher relative abundance of the ASVs in sediment samples than in the water samples suggests that these bacteria are more likely to be concentrated in the sediment. If pathogenic, these bacteria could potentially infect corals through direct contact or indirectly by moving into the water column surrounding the colonies. The results of the present study suggest that additional research into the roles of *Rhodobacterales* and *Rhizobiales* in SCTLD lesions and of sediment as a source of transmission will further the understanding of the SCTLD outbreak.

## Data Availability Statement

The datasets generated and analyzed during the current study are available on NCBI under BioProject number PRJNA576217. The code and data files for this study are available on GitHub at https://github.com/srosales712/SCTLD_16S_2019.

## Author Contributions

EM, LH, and RR conceived of the collection design and provided funding. LH, AC, and RR collected the samples. AC processed the samples. SR analyzed the microbiome data. SR, AC, and LH made the tables and figures. SR wrote the manuscript with assistance from LH and AC. All authors edited the manuscript and assisted with interpretation of the results.

## Conflict of Interest

The authors declare that the research was conducted in the absence of any commercial or financial relationships that could be construed as a potential conflict of interest.

## Footnotes

1 www.mrdnalab.com

2 http://www.mrdnafreesoftware.com/fasta-qual-fastq-conversion.html
