# Supplementary material for: Rhodobacterales and Rhizobiales Are Associated With Stony Coral Tissue Loss Disease and Its Suspected Sources of Transmission
Source: Front Microbiol. 2020 Apr 23;11:681. doi: 10.3389/fmicb.2020.00681 (PMC7212369; doi:10.3389/fmicb.2020.00681)
Supplement: Supplementary file 1 [file Data_Sheet_1.PDF]

Title: *Rhodobacterales* and *Rhizobiales* are associated with Stony Coral Tissue Loss Disease and its suspected sources of transmission

Authors: Stephanie M. Rosales [1,2*], Abigail S. Clark[3], Lindsay K. Huebner[4], Rob R. Ruzicka[4], Erinn M. Muller[5]

Affiliations:

1. Cooperative Institute for Marine and Atmospheric Studies, University of Miami, Miami, Florida, USA.
2. Atlantic Oceanographic and Meteorological Laboratory, National Oceanographic and Atmospheric Administration, Miami, Florida, USA
3. Elizabeth Moore International Center for Coral Reef Research & Restoration, Mote Marine Laboratory, Summerland Key, Florida, USA 33042
4. Fish & Wildlife Research Institute, Florida Fish & Wildlife Conservation Commission, St. Petersburg, Florida, USA 33701
5. Mote Marine Laboratory, Sarasota, Florida, USA 34236

* Correspondence Author email: Stephanie.Rosales@noaa.gov

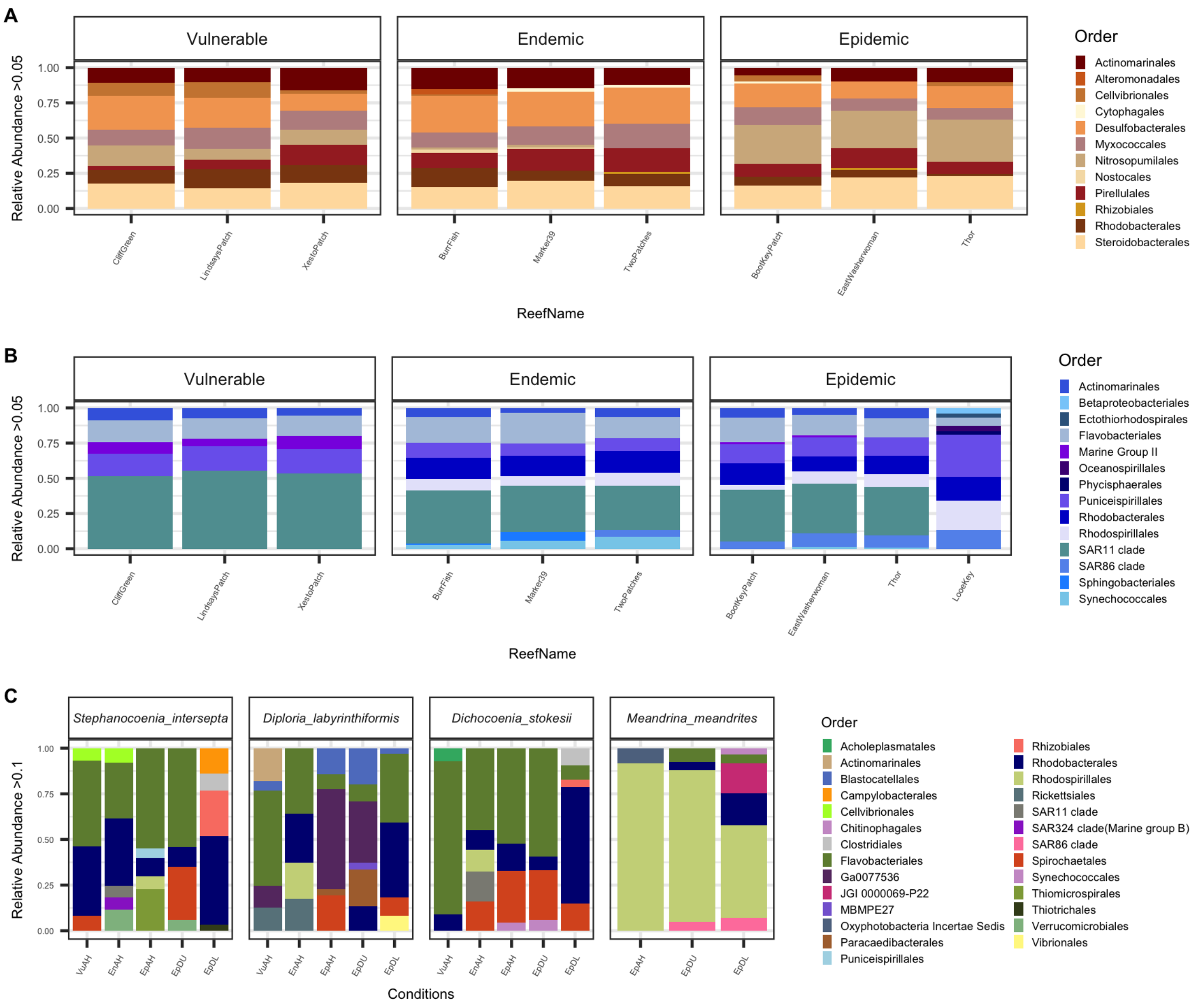

Supplementary Figure 1. Sediment, water, and coral tissue samples show distinct dominant microbial taxa. The cumulative percent relative abundances of the most abundant microbial orders in (A) sediment (>0.05%), (B) water (>0.05%), and (C) tissue (>0.01%) samples. Each stacked color bar represents a different order. A-B are grouped by zone (vulnerable, endemic, epidemic) and reef name. In C, the data are grouped by coral species, zone, and tissue condition (vulnerable zone apparently healthy coral [VuAH], endemic zone apparently healthy coral [EnAH], epidemic zone apparently healthy coral [EpAH], epidemic zone unaffected area on a diseased coral [EpDU], and epidemic zone lesion tissue on a diseased coral [EpDL]). Sample sizes are provided in Table 1.

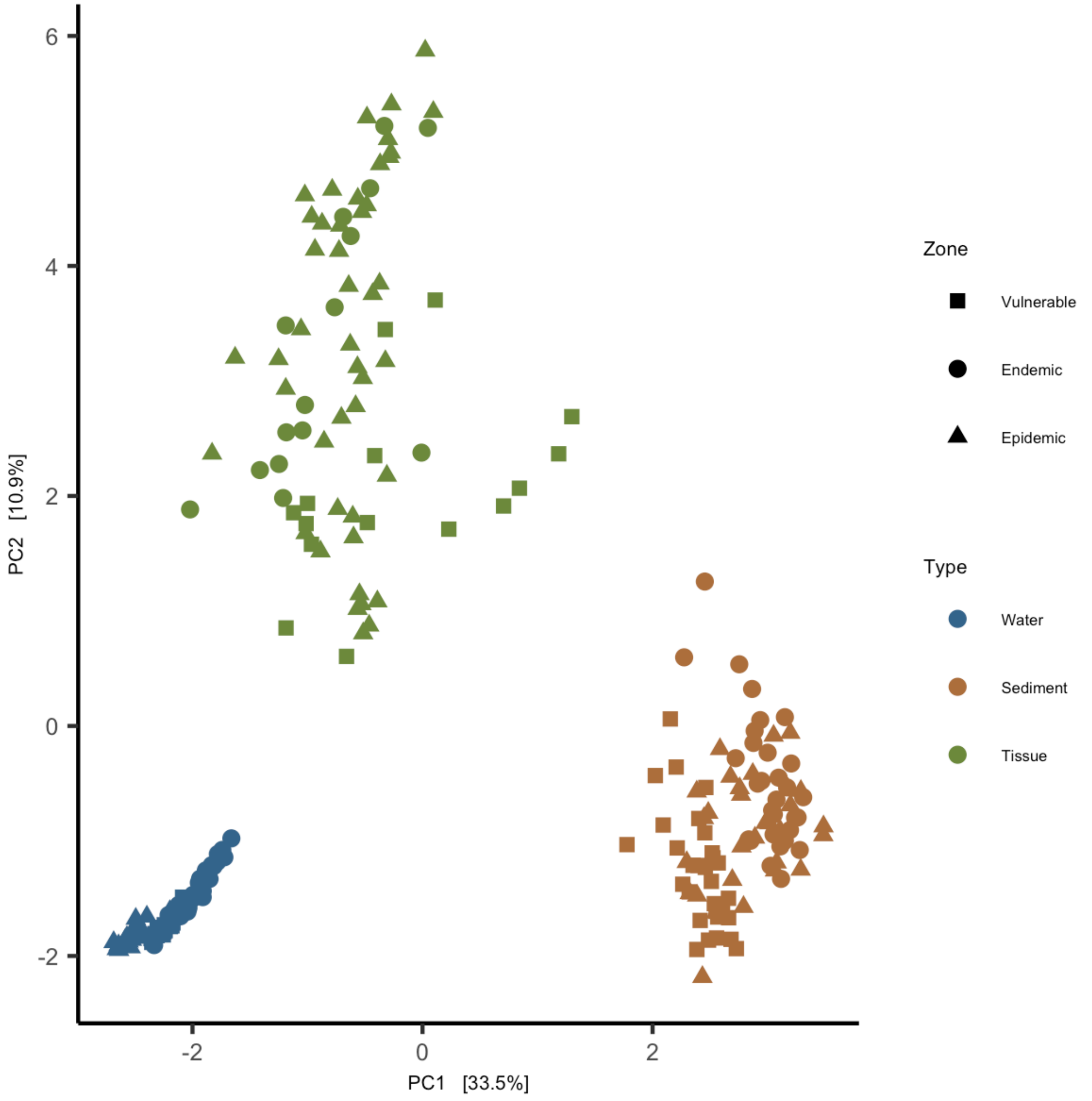

Supplementary Figure 2. Sediment, water, and coral tissue samples show distinct microbial beta-diversity. Principal component analysis (PCA) with a Euclidean distance of sediment, water, and coral tissue sample types (colors). Shapes represent the three zones: vulnerable (square), endemic (circle), and epidemic (triangle).

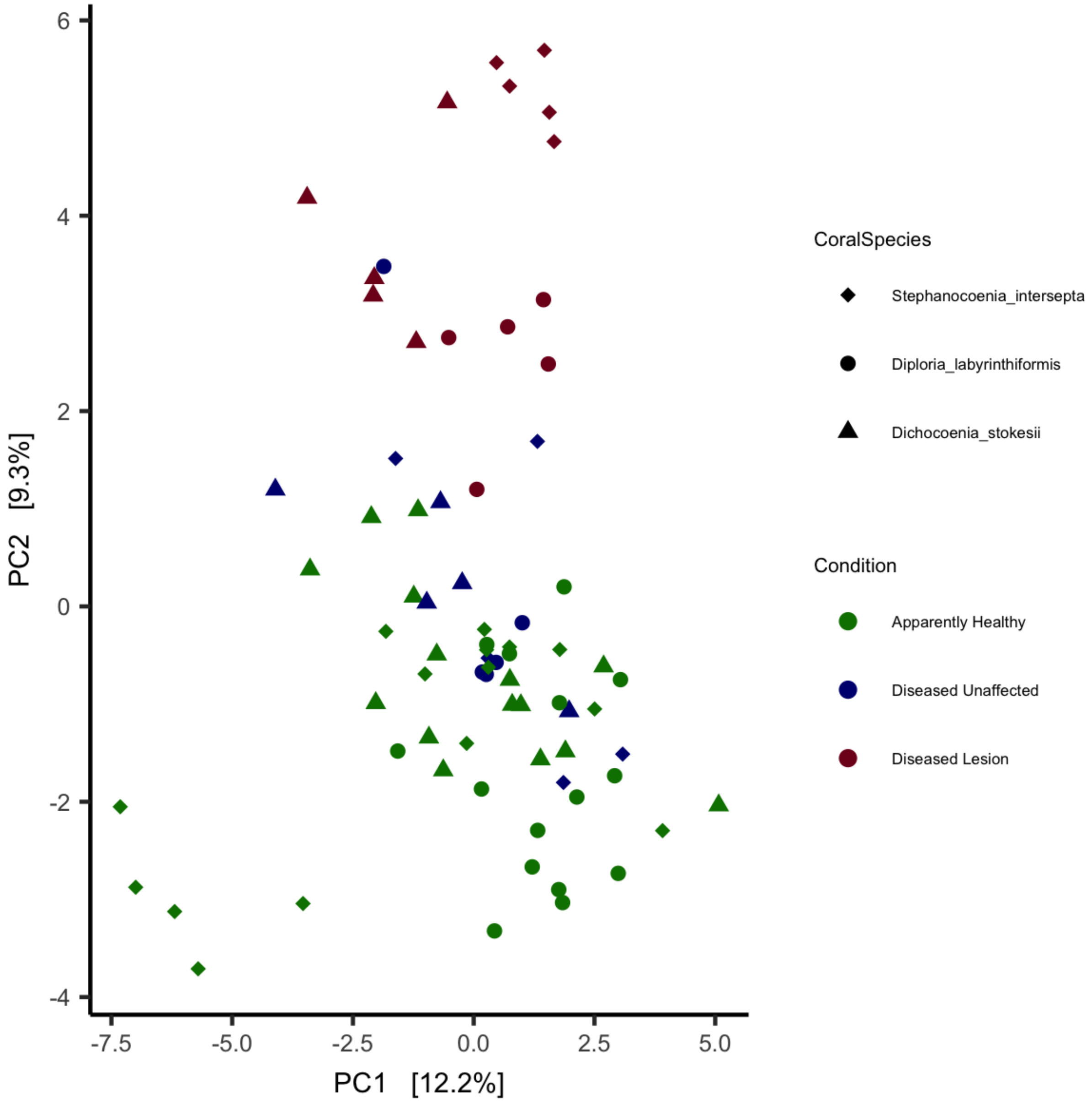

Supplementary Figure 3. Corals show distinct microbial beta-diversity by tissue condition. Principal component analysis (PCA) with a Euclidean distance. Each shape represents a different coral species: *Stephanocoenia intersepta* (diamond), *Diploria labyrinthiformis* (circle), and *Dichocoenia stokesii* (triangle). Shapes are colored by apparently healthy colony tissue (green), and unaffected tissue (blue) and lesion tissue (red) from diseased colonies.
